# Gender and racial/ethnic differences in the associations of urinary phthalate metabolites with markers of diabetes risk: national health and nutrition examination survey 2001–2008

**DOI:** 10.1186/1476-069X-13-6

**Published:** 2014-02-05

**Authors:** Tianyi Huang, Aditi R Saxena, Elvira Isganaitis, Tamarra James-Todd

**Affiliations:** 1 Division of Women’s Health, Department of Medicine, Connors Center for Women’s Health and Gender Biology, Brigham and Women’s Hospital and Harvard Medical School, Boston, MA 02120, USA; 2 Department of Epidemiology, Harvard School of Public Health, Boston, MA 02115, USA; 3 Division of Endocrinology, Department of Medicine, Brigham and Women’s Hospital and Harvard Medical School, Boston, MA 02115, USA; 4 Genetics and Epidemiology Division and Pediatric Health Services, Joslin Diabetes Center, Boston, MA 02215, USA

**Keywords:** Di(2-ethylhexyl) phthalate, Mono-benzyl phthalate, Mono-(3-carboxypropyl) phthalate, Mono-ethyl phthalate, Mono-isobutyl phthalate, Mono-n-butyl phthalate, Insulin, Blood glucose, Gender differences, Race/ethnicity

## Abstract

**Background:**

Phthalates are ubiquitous endocrine disrupting chemicals associated with diabetes. Although women and minorities are more likely to be exposed to phthalates, no prior studies have examined phthalate exposure and markers of diabetes risk evaluating effect modification by gender and race/ethnicity.

**Methods:**

We analyzed CDC data for 8 urinary phthalate metabolites from 3,083 non-diabetic, non-pregnant participants aged 12- < 80 years in the National Health and Nutrition Examination Survey (NHANES) 2001–2008. We used median regression to assess the associations between urinary phthalate metabolites and fasting blood glucose (FBG), fasting insulin and Homeostatic Model Assessment of insulin resistance (HOMA-IR), controlling for urinary creatinine as well as several sociodemographic and behavioral factors. Stratified analyses were conducted to compare the gender- and race/ethnicity-specific patterns for the associations.

**Results:**

Urinary levels of several phthalate metabolites, including MBzP, MnBP, MiBP, MCPP and ∑DEHP showed significant positive associations with FBG, fasting insulin and HOMA-IR. No clear difference was noted between men and women. Mexican-Americans and non-Hispanic blacks had stronger dose–response relationships for MnBP, MiBP, MCPP and ∑DEHP compared to non-Hispanic whites. For example, the highest quartile of MiBP relative to its lowest quartile showed a median FBG increase of 5.82 mg/dL (95% CI: 3.77, 7.87) in Mexican-Americans, 3.63 mg/dL (95% CI: 1.23, 6.03) in blacks and 1.79 mg/dL (95% CI: -0.29, 3.87) in whites.

**Conclusions:**

The findings suggest that certain populations may be more vulnerable to phthalates with respect to disturbances in glucose homeostasis. Whether endocrine disrupting chemicals contribute to gender and racial/ethnic differences in diabetes risk will be an important area for further study.

## Background

Phthalates are a family of diester compounds of 1,2-benzenedicarboxylic acid widely used as plasticizers, solvents or additives. They are found in cosmetics, food wrapping, medical devices and a large number of other consumer products [1-3]. Human exposure is detectable in urine in >75% of the U.S. population [4] and is mostly due to the absorption, inhalation or ingestion of phthalates from these products [5,6]. Moreover, phthalates have been shown to have developmental and reproductive toxicity in experimental animals [7] and have raised public health concerns for a variety of adverse health outcomes, notably genitourinary malformations and infertility related to anti-androgen effects [8,9], in humans.

An emerging body of evidence suggests that phthalates may interfere with glucose homeostasis and insulin sensitivity, leading to an increased risk of diabetes [10-13]. Phthalates have been demonstrated to bind to peroxisome proliferator-activated receptors (PPARs) [14,15], a family of nuclear receptors that controls lipid storage and carbohydrate metabolism [16-18]. Interactions between phthalates and PPAR-gamma may contribute to dysregulation of glucose metabolism and promotion of adipogenesis [7,17,19]. Moreover, the predisposition to obesity and diabetes may be influenced by in utero exposure to phthalates [20,21]. Although a positive association has been reported in US males between specific phthalates and insulin resistance, as assessed by Homeostatic Model Assessment of insulin resistance (HOMA-IR) [22], no studies to our knowledge have examined gender and racial/ethnic differences in associations between phthalates and markers of glucose homeostasis and insulin resistance (hereafter ‘markers of diabetes risk’).

The importance of evaluating this research question lies in population studies showing higher insulin resistance and increased risk of type 2 diabetes in women versus men and in blacks and Hispanics compared with whites [23-26]. While genetic and lifestyle factors have been examined [27,28], few published studies have investigated environmental factors, such as phthalate exposure, as potential contributors to the increased risk of type 2 diabetes in these populations. Interestingly, several studies have found urinary phthalate metabolite levels to be higher in women and non-white ethnic groups [5,29]. As such, it is essential to examine how the nearly ubiquitous phthalate exposure in the U.S. population may contribute to gender and racial/ethnic differences in glucose regulation, insulin resistance and diabetes risk.

In this exploratory study, we hypothesize that increased levels phthalate metabolites are associated with higher levels of markers of diabetes risk in men and women without the diagnosis of diabetes. Furthermore, we posit that gender and race/ethnicity may modify the positive associations. We examined the associations of 8 urinary phthalate metabolites with three markers of diabetes risk including fasting blood glucose (FBG), fasting insulin and HOMA-IR in participants of the National Health and Nutrition Examination Survey (NHANES) 2001–2008, and compared the patterns of these associations across gender and racial/ethnic subgroups with evaluations of potential interactions.

## Methods

### Study participants

For this analysis we pooled data from 2001–2008 NHANES, a nationally representative survey conducted by the National Center for Health Statistics (NCHS), Centers of Disease Control and Prevention (CDC) to assess the health and nutritional status among the civilian, non-institutionalized US population. A complex, multistage, probability sampling strategy was used, with oversampling of subgroups of particular public health interest, including individuals of lower socioeconomic status and racial/ethnic minorities.

The current analysis included men and women aged 12- < 80 years who did not carry the diagnosis of diabetes, as the analysis suggested no differences between adolescents (12–20 years) and adults in the associations of phthalates with markers of diabetes risk. Diabetes diagnosis was based on participants’ answer to the question: “Other than during pregnancy, have you ever been told by a doctor or health professional that you have diabetes or sugar diabetes?” We included only those participants who responded ‘no’ to self-reported diabetes. For women of reproductive age (defined by NHANES as 20–44 years for 2007–2008 cycle and 12–59 years for all other cycles), only those with confirmed negative urine pregnancy tests were included in this analysis.

### Measurements of phthalate exposure

Urinary phthalate metabolites were measured in a random, one-third subsample of NHANES participants. Urine samples were collected and stored at -20°C before being shipped to CDC's National Center for Environmental Health for analysis. Laboratory testing results below limits of detection (LOD) were replaced with LOD divided by the square root of 2 [30]. Details regarding phthalate measurements can be found elsewhere [5].

We selected the following 8 phthalate metabolites that were measured in all NHANES cycles from 2001 to 2008: mono-ethyl phthalate (MEP), mono-n-butyl phthalate (MnBP), mono-isobutyl phthalate (MiBP), mono-benzyl phthalate (MBzP), mono-(3-carboxypropyl) phthalate (MCPP), mono-(2-ethyl-hexyl) phthalate (MEHP), mono-(2-ethyl-5-hydroxyhexyl) phthalate (MEHHP), and mono-(2-ethyl-5-oxohexyl) phthalate (MEOHP). The latter three metabolites of di-2-ethylhexyl phthalates (DEHP), MEHP, MEHHP and MEOHP, were combined into one measure in molar concentrations for analysis, denoted by ∑DEHP. Phthalate metabolites were divided into quartiles with the lowest category as the reference level. To account for the influence of urine volume and renal function on phthalate measurement, we reported creatinine-adjusted levels by dividing the phthalate concentration by urinary creatinine concentration.

### Markers of diabetes risk

Fasting blood samples were collected during the morning examination session with measurement of FBG (mg/dL) and insulin levels (uU/mL). Among the study participants, 6.4% were fasted <8 hours at the time of blood sample collection, and 3.2% had either FBG > 126 mg/dL or hemoglobin A1c > 6.5%. Since exclusions of these potential outliers did not change the results materially, we kept them in the analysis with appropriate statistical manipulations (described below) to minimize their possible influences. To quantify insulin resistance, we calculated Homeostatic Model Assessment of insulin resistance (HOMA-IR) with the following equation [31]:

$$\mathrm{HOMA}=\frac{\mathrm{Fasting}\;\mathrm{glu}\cos e\;\left(\mathrm{mml}/L\right)\;\times \;\mathrm{Fasting}\;\mathrm{insulin}\;\left(\mathrm{uU}/\mathrm{ml}\right)}{22.5}$$

FBG, insulin and HOMA-IR were all evaluated as continuous outcomes.

### Covariates

Covariates used in the analysis include age, gender, race/ethnicity, fasting time, urinary creatinine, total caloric intake, triglyceride, smoking status, education and poverty. Total fat intake and physical activity were also considered but not used in analysis, as they did not change the effect estimates substantially or contribute to the models as strong predictors. Age was categorized as 12- < 20, 20- < 60 and 60- < 80 years. Self-identified race/ethnicity was categorized as non-Hispanic white, non-Hispanic black, Mexican-Americans and other. To account for the variations in fasting time, as well as the small proportion of participants who fasted < 8 hour, we created a single continuous measure for fasting time.

Total caloric intake, estimated by 24-hour dietary recall in 2001–2002 and by the average caloric intake from the 2-day recall in 2003–2008, was categorized into quartiles. We created quartiles for triglyceride levels. Smoking status was defined as never smoker, current smoker and past smoker, with the addition of an “N/A” category for participants under 20 for whom data on smoking history were not available. We evaluated education as high school graduate or less, some college, college graduate or higher and unfinished education for participants under 20. Poverty was determined based on income-to-poverty ratio, with the ratio ≤ 1 being under the poverty level [32].

Body mass index (BMI) may be an important intermediate or confounding variable for the associations under investigation. We divided BMI into four categories: underweight, normal weight, overweight, and obese, using BMI cutoff points (<18.5, 18.5-25, 25–30 and ≥30 kg/m^2^) for adults and percentile cutoff points (<5^th^, 5^th^-85^th^, 85^th^-95^th^, and ≥95^th^ percentile) for adolescents [33].

### Statistical analysis

We conducted a complete-subject analysis based on eligible subjects without missing covariate information. Population characteristics across gender and racial/ethnic categories (non-Hispanic white, non-Hispanic black, Mexican-American and other) were presented in medians and interquartile ranges.

We used median regression to reduce the impact of the non-normal, highly-skewed diabetes biomarker levels, allowing the robust modeling of continuous outcomes on their measured scale [34-36]. The analyses were conducted separately for each phthalate. Median regression was performed for each of the markers of diabetes risk across phthalate quartiles based on raw concentrations, adjusted for age, gender, race/ethnicity, fasting time, urinary creatinine [37], total caloric intake, triglycerides, education, poverty and smoking status in all phthalate models. The coefficient for a given phthalate quartile represents the median change of the markers of diabetes risk comparing that phthalate quartile to the lowest quartile after accounting for the other factors in the model. Markov Chain Marginal Bootstrap (MCMB) re-sampling method was used to compute 95% confidence intervals (95% CI), and p-values for trend across phthalate quartiles were reported by using the median value in each category as a single continuous variable. Multiple comparisons were not adjusted for in the analysis, given the exploratory nature of the study [38]. We also ran models that further included BMI, which could not be ruled out as potential confounders or intermediates given the cross-sectional design.

To evaluate the gender and racial/ethnic differences in the associations between phthalates and markers of diabetes risk, we conducted stratified analyses using the same model in the two gender groups (males and females) and the three major racial/ethnic groups (white, black and Mexican-American). ‘Other’ race/ethnicity group was not considered due to its small sample size and mixed composition. We used the quartiles derived from the phthalate distribution of the overall population in the stratified analysis. We also repeated the analysis by creating gender- and race/ethnicity-specific quartiles for each phthalate. Associations were slightly stronger, but for consistency, we reported the results based on overall-population quartiles. In addition, we added cross-product terms to evaluate the effect modification by gender and race/ethnicity, respectively, interpreting p < 0.10 from likelihood ratio test as a significant interaction. All analyses were conducted in SAS 9.2 (Cary, NC).

## Results

### Population characteristics

Of 3,870 participants between ages 12 and <80 having data on urinary phthalate metabolites, fasting glucose and insulin levels, we excluded n = 322 people who self-reported ‘yes’ or ‘borderline’ for diabetes diagnosis status, refused, or had missing information. We additionally excluded n = 146 women of reproductive age who had positive, invalid or missing pregnancy tests or who were eligible but had not completed the tests. Another n = 3 current insulin users were also excluded, which left n = 3,399. Of these, n = 316 subjects were excluded as a result of missing covariates, resulting in a total of n = 3,083 study participants for this analysis.

Women had higher creatinine-adjusted concentrations for all six selected phthalate metabolites than men (Table 1). Men had higher FBG levels, but lower fasting insulin levels, which contributed to slightly smaller measures of HOMA-IR compared to women. Levels of phthalate metabolites also varied across the three major ethnic groups, with blacks having the highest levels of MEP, MnBP, MiBP, MBzP, and ∑DEHP and whites having the highest MCPP levels. Mexican-Americans had higher urinary concentrations of MnBP and MiBP than whites. With regard to the markers of diabetes risk, Mexican-Americans and blacks had comparable levels of fasting insulin, but Mexican-Americans had higher levels of FBG. Whites had similar levels of FBG to African-Americans, but lower fasting insulin levels. Mexican-Americans yielded the highest measures of HOMA-IR, while whites were lowest in HOMA-IR.

**Table 1:** Study population characteristics across gender and racial/ethnic groups, NHANES 2001-2008

	**Gender**		**Race/ethnicity**			
	**Men (n = 1620)**	**Women (n = 1463)**	**White (n = 1362)**	**Black (n = 719)**	**Mexican-American (n = 726)**	**Other (n = 276)**
	Median (interquartile range)					
Phthalate metabolites^*^						
MEP	125.3	181.9	122.3	190.4	186.7	141.8
	(57.8, 340.9)	(89.0, 425.6)	(55.0, 325.7)	(88.2, 428.1)	(86.6, 446.4)	(64.0, 346.6)
MBzP	10.4	13.4	11.5	13.1	11.0	12.0
	(5.4, 19.5)	(7.1, 23.8)	(6.0, 20.9)	(7.2, 23.6)	(5.5, 20.4)	(6.1, 23.3)
MnBP	13.6	22.3	16.0	18.4	17.4	19.6
	(8.7, 22.3)	(13.2, 35.9)	(9.6, 26.3)	(11.2, 28.6)	(10.5, 29.9)	(12.0, 32.6)
MiBP	3.8	4.9	3.5	5.0	4.6	6.4
	(2.0, 6.6)	(2.6, 8.9)	(1.8, 6.1)	(2.8, 8.8)	(2.4, 8.0)	(3.3, 11.0)
MCPP	2.0	2.3	2.3	1.9	2.2	2.1
	(1.2, 3.3)	(1.4, 4.0)	(1.4, 3.9)	(1.1, 3.2)	(1.3, 3.7)	(1.3, 3.9)
∑DEHP	9.5	11.5	10.2	11.4	9.8	11.4
	(5.3, 19.7)	(6.5, 23.1)	(5.7, 22.6)	(5.8, 23.2)	(5.8, 17.8)	(7.1, 22.3)
Markers of diabetes risk						
Fasting glucose (mg/dL)	96.9	93.0	96.0	92.0	95.7	95.0
	(91.0, 104.0)	(88.0, 100.0)	(90.1, 103.2)	(87.0, 99.0)	(90.7, 102.0)	(90.0, 102.0)
Fasting insulin (uU/mL)	9.3	9.9	8.6	10.4	10.8	8.9
	(6.0, 14.4)	(6.4, 15.2)	(5.6, 13.2)	(6.5, 17.1)	(7.1, 16.3)	(5.9, 13.9)
HOMA-IR	2.2	2.3	2.1	2.3	2.6	2.1
	(1.4, 3.6)	(1.4, 3.7)	(1.3, 3.3)	(1.4, 4.0)	(1.7, 4.0)	(1.4, 3.3)
Selected covariates						
Age (yrs)	33	34	42	24	25	32
	(18, 52)	(18, 51)	(23, 59)	(16, 46)	(16, 44)	(18, 49)
BMI (kg/m^2^)^**^	26.0	26.5	26.1	26.0	26.4	25.6
	(22.2, 29.8)	(22.1, 31.6)	(22.6, 30.3)	(21.8, 31.8)	(22.1, 30.3)	(21.7, 29.2)
Total caloric intake (kcal)	2399	1720	2101	1972	2028	1903
	(1845, 3099)	(1342, 2187)	(1591, 2764)	(1459, 2680)	(1546, 2614)	(1401, 2617)
Creatinine (mg/dL)	147	116	125	165.0	124.0	132
	(103, 208)	(72, 173)	(75, 176)	(110, 234)	(84, 175)	(89, 185)

^*^Creatinine-adjusted phthalates: μg/g creatinine for MEP, MBzP, MnBP, MiBP and MCPP; μmol/100 g creatinine for ∑DEHP.

^**^Number of subjects for BMI is slightly different due to missing values: male (1607), female (1447); white (1352), black (710), Mexican-American (710), other (271).

### Phthalate metabolites and markers of diabetes risk in the overall population

Associations of each diabetes biomarker across quartiles of phthalates for those without self-reported diabetes are presented in Table 2. Positive associations were observed for MnBP, MiBP, MCPP and ∑DEHP with FBG, fasting insulin and HOMA-IR. MiBP was most strongly associated with FBG, with a median increase of 1.87 (95% CI 0.83-2.92), 2.77 (95% CI 1.75-3.80) and 3.69 (95% CI 2.60-4.78) mg/dL in quartiles 2–4, respectively, compared to quartile 1 (p-trend < 0.0001). Compared to the lowest quartile, higher quartiles of ∑DEHP were associated with higher fasting insulin levels (p-trend < 0.0001). ∑DEHP also had the most significant associations with HOMA-IR; the median values were 0.49 (95% CI 0.31, 0.66), 0.51 (95% 0.34, 0.69) and 0.68 (95% CI 0.47, 0.88) units higher with increasing quartiles of metabolite concentrations compared to the lowest quartile (p-trend < 0.0001). While most phthalates showed a strong positive association with all three markers of diabetes risk, MBzP was not associated with FBG and MEP did not show statistically significant associations with any of the markers of diabetes risk. Adjustment for BMI modestly attenuated almost all associations (results not shown).

**Table 2:** Associations of phthalates with markers of diabetes risk in the overall population, NHANES 2001-2008

Phthalate metabolites^*^	**Biomarkers for diabetes**		
	**Fasting glucose**	**Fasting insulin**	**HOMA-IR**
	Median change (95% CI)^**^		
MEP			
Q1	Ref.	Ref.	Ref.
Q2	1.30 (0.15, 2.46)	0.25 (-0.50, 0.99)	0.11 (-0.06, 0.27)
Q3	0.38 (-0.75, 1.51)	0.34 (-0.35, 1.02)	0.10 (-0.07, 0.28)
Q4	0.49 (-0.80, 1.77)	0.60 (-0.13, 1.34)	0.20 (0.03, 0.38)
p for trend	0.7589	0.1480	0.0477
MBzP			
Q1	Ref.	Ref.	Ref.
Q2	-0.30 (-1.48, 0.87)	0.77 (0.16, 1.39)	0.21 (0.06, 0.37)
Q3	-0.06 (-1.25, 1.13)	1.09 (0.39, 1.79)	0.26 (0.09, 0.44)
Q4	-0.24 (-1.49, 1.02)	1.44 (0.50, 2.38)	0.37 (0.15, 0.59)
p for trend	0.7058	0.0070	0.0028
MnBP			
Q1	Ref.	Ref.	Ref.
Q2	0.95 (-0.22, 2.13)	1.15 (0.52, 1.78)	0.28 (0.11, 0.44)
Q3	1.70 (0.51, 2.89)	1.41 (0.72, 2.09)	0.28 (0.11, 0.46)
Q4	1.91 (0.51, 3.31)	1.11 (0.31, 1.92)	0.34 (0.15, 0.54)
p for trend	0.0193	0.0918	0.0059
MiBP			
Q1	Ref.	Ref.	Ref.
Q2	1.87 (0.83, 2.92)	1.45 (0.85, 2.04)	0.38 (0.23, 0.52)
Q3	2.77 (1.75, 3.80)	1.23 (0.57, 1.89)	0.35 (0.19, 0.51)
Q4	3.69 (2.60, 4.78)	1.73 (0.92, 2.54)	0.53 (0.33, 0.72)
p for trend	<0.0001	0.0028	0.0002
MCPP			
Q1	Ref.	Ref.	Ref.
Q2	0.72 (-0.44, 1.89)	0.90 (0.24, 1.55)	0.23 (0.06, 0.40)
Q3	0.94 (-0.34, 2.21)	1.31 (0.59, 2.03)	0.34 (0.16, 0.52)
Q4	2.42 (1.22, 3.62)	0.94 (0.12, 1.77)	0.28 (0.07, 0.48)
p for trend	<0.0001	0.0629	0.0269
∑DEHP			
Q1	Ref.	Ref.	Ref.
Q2	1.47 (0.30, 2.63)	1.74 (1.04, 2.45)	0.49 (0.31, 0.66)
Q3	1.75 (0.66, 2.84)	1.99 (1.31, 2.66)	0.51 (0.34, 0.69)
Q4	2.45 (1.29, 3.60)	2.60 (1.82, 3.38)	0.68 (0.47, 0.88)
p for trend	0.0016	<0.0001	<0.0001

^*^All analyses adjusted for age, sex, race, urinary creatinine, fasting time, total caloric intake, triglyceride, education, smoking status and poverty.

^**^Fasting glucose: mg/dL, fasting insulin: uU/mL.

### Phthalate metabolites and markers of diabetes risk by gender

**Figure 1:**
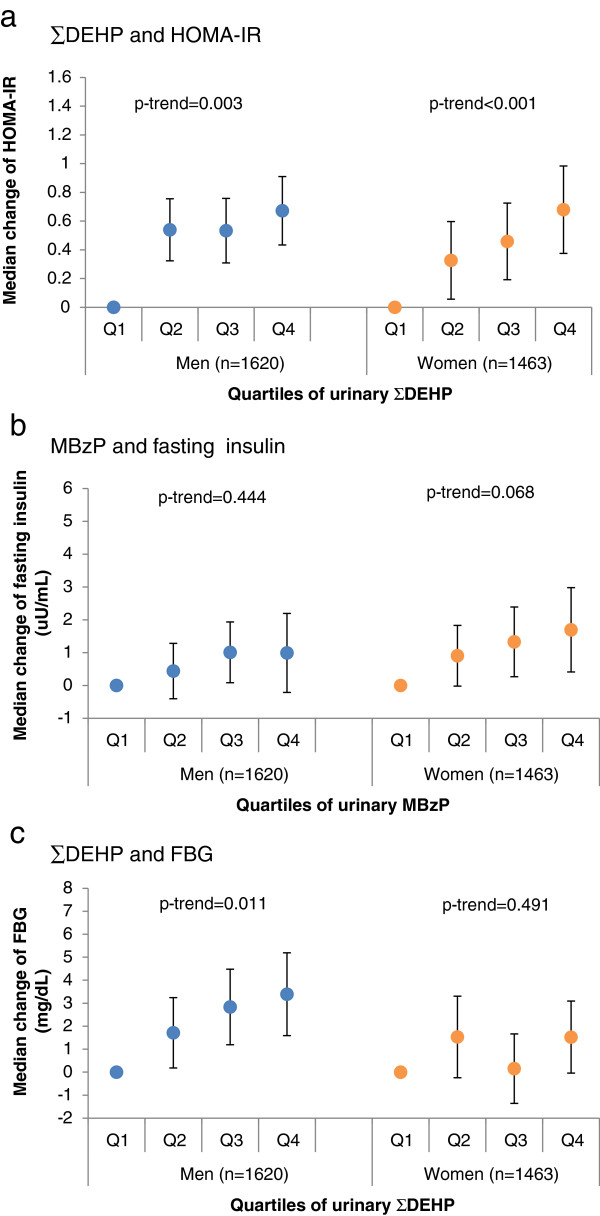
**Change in markers of diabetes risk across quartiles of markers of diabetes risk in men and women.** One example is selected for each pattern. **(a)** example of similar strength of association: ∑DEHP and HOMA-IR, most associations show similar gender patterns, **(b)** example of stronger association in women: MBzP and fasting insulin, **(c)** example of stronger association in men: ∑DEHP and FBG. Adjusted for age, race, urinary creatinine, fasting time, total caloric intake, triglyceride, education, smoking status and poverty for all phthalate metabolites.

Most of the positive associations that had previously been observed in the overall population were also seen in gender subgroups. For the majority of these associations, the strengths were similar in men and women, although the exact dose–response patterns varied by gender. For example, the fourth quartile of ∑DEHP was associated with a HOMA-IR increase of 0.67 (95% CI 0.43, 0.91) in men and 0.68 (95% CI 0.38, 0.98) in women (Figure 1-a), but there seemed to be a maximal dose–response plateau in men, while the increasing trend was appreciably linear in women (p for interaction = 0.06). Notably, the strengths of three associations were suggestively different by gender group. For example, stronger associations were seen in women for MBzP with fasting insulin (p for interaction = 0.02), with the fourth quartile conferring a 1.69 (95% CI 0.41, 2.98) uU/mL increase in women compared to a 0.99 (95% CI -0.21, 2.19) uU/mL increase in men (Figure 1-b). On the other hand, men appeared to have stronger associations for ∑DEHP and FBG than women (p for interaction = 0.04, Figure 1-c). However, these potential gender differences were not consistently observed across phthalate metabolites or across markers of diabetes risk. The complete results were given in Additional file 1.

### Phthalate metabolites and markers of diabetes risk by race/ethnicity

**Figure 2:**
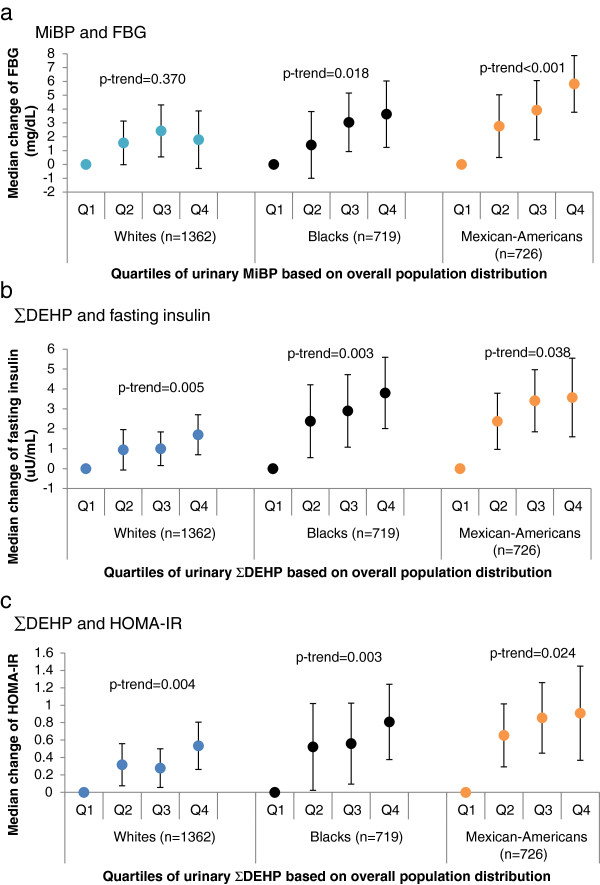
**Change in markers of diabetes risk across quartiles of urinary phthalate metabolites in racial/ethnic subgroups.** One example is selected for each diabetes biomarker, and similar racial/ethnic patterns were seen for other urinary phthalate metabolites, particularly MnBP, MiBP, MCPP and ∑DEHP. **(a)** example of FBG: MiBP and FBG, **(b)** example of fasting insulin: ∑DEHP and fasting insulin, **(c)** example of HOMA-IR: ∑DEHP and HOMA-IR. Adjusted for age, sex, urinary creatinine, fasting time, total caloric intake, triglyceride, education, smoking status and poverty for all phthalate metabolites.

For MnBP, MiBP, MCPP and ∑DEHP, the positive associations with FBG appeared to be strongest in Mexican-Americans, intermediate in blacks, and weakest in whites. For example, compared to the lowest quartile, the highest quartile of MiBP was associated with a median FBG increase of 5.82 (95% CI 3.77, 7.87, p-trend < 0.01) mg/dL in Mexican-Americans, a smaller increase of 3.63 (95% CI 1.23, 6.03, p-trend = 0.02) mg/dL in blacks and a non-significant increase of 1.79 (95% CI -0.29, 3.89, p-trend = 0.37) mg/dL in whites (Figure 2-a). Similar racial patterns were consistently seen for MnBP, MCPP and ∑DEHP. No significant associations were present for MEP or MBzP in any racial/ethnic category.

Depending on the type of phthalate metabolites, either Mexican-Americans or blacks showed stronger dose–response associations with fasting insulin compared to whites. Significant monotonic positive trends were observed for ∑DEHP in all three racial/ethnic groups, and the associations between ∑DEHP and fasting insulin appeared to be larger in magnitude among blacks and Mexican-Americans than among whites (Figure 2-b). There was a suggestion of a strong non-monotonic association for MnBP and fasting insulin only in Mexican Americans, with those in the third quartile being 3.04 uU/mL higher (95% CI 1.51, 4.57). On the other hand, MiBP had a monotonically increasing pattern only in Mexican-Americans (p-trend = 0.15) and MCPP had a similar pattern only in blacks (p-trend = 0.01), whereas these two phthalates had non-monotonic and non-significant positive associations with fasting insulin among whites. Positive, but weaker associations were seen for MBzP and insulin levels across all racial/ethnic groups. There did not appear to be any significant associations for MEP and insulin levels.

The race-specific associations between HOMA-IR and phthalate metabolites revealed almost the same patterns as reported for fasting insulin. Of all six phthalate metabolites, ∑DEHP was associated with the strongest elevations of HOMA-IR across three racial groups, with the fourth quartile conferring a median increase of 0.81 (95% CI 0.38, 1.24) in blacks, 0.91 (95% CI 0.37, 1.45) in Mexican-Americans and 0.53 (95% CI 0.26, 0.81) in whites compared to the lowest quartile (Figure 2-c). The associations with HOMA-IR were also strongest in blacks or Mexican-Americans for the other five phthalate metabolites except MBzP, the fourth quartile of which was associated with the greatest increase of 0.39 (95% CI 0.09, 0.69) in whites. However, despite the suggestively stronger dose–response associations in blacks and Mexican-Americans, we did not find statistically significant interactions by race/ethnicity for any phthalate metabolite in relation to the three biomarkers, except for the interaction with MnBP for HOMA-IR (p for interaction = 0.09). The complete results were given in Additional file 2.

## Discussion

This exploratory analysis of NHANES 2001–2008 suggests that higher levels of certain phthalate metabolites were associated with elevated FBG, fasting insulin and insulin resistance, with some variations by race/ethnicity and less by gender. Specifically, among the phthalate metabolites examined, MnBP, MiBP, MCPP and ∑DEHP exhibited strong positive associations with FBG, fasting insulin and HOMA-IR. MBzP was associated with fasting insulin and HOMA-IR only. The strength of associations between levels of phthalate metabolites and markers of diabetes risk appeared to be mostly the same by gender. By contrast, we noted differences in associations by racial/ethnic group, with non-Hispanic blacks and Mexican-Americans having stronger associations than non-Hispanic whites. These results may suggest that phthalates could be involved in altering glucose homeostasis and insulin sensitivity, with certain populations being more vulnerable.

Our study found similar associations as a recent study of adult women in the NHANES 2001–2008 [10], which showed that MiBP and ∑DEHP were associated with increased insulin resistance. However, MnBP and MCPP were not found to be positively associated with either fasting glucose or HOMA-IR in that study, possibly due to the restrictions by sample size. Also, the present study showed stronger dose–response associations. A study of only male participants from NHANES 1999–2002 found that MBzP, MnBP and MEP were associated with increased HOMA-IR [22], whereas our results revealed non-significant positive associations for MEP.

The present study adds to the growing body of literature showing phthalates being positively associated with diabetes and its risk factors [10-12,22]. One way that phthalates could operate to alter normal glucose metabolism among individuals without diabetes is through their ability to bind to PPAR-alpha and PPAR-gamma [14,15]. While PPAR-gamma agonists have great therapeutic potential in the treatment of type 2 diabetes for their potent insulin-sensitizing activity and anti-diabetic effects [18], it is unclear whether phthalates modulate PPAR-gamma in the same way. As such, more studies are needed to determine the consequences of phthalates binding to PPAR in humans, particularly with respect to selective PPAR-gamma modulation [39]. Furthermore, more research needs to be conducted on PPAR-alpha and its response to phthalates.

Although we observed similar strengths in the associations between a majority of phthalate metabolites and markers of diabetes risk, a few exceptions seem to show stronger associations in women (e.g. MiBP/fasting insulin and MBzP/fasting insulin) or in men (e.g., ∑DEHP/FBG and MCPP/HOMA-IR). Although these few gender differences may be chance findings due to inflated type I errors from large number of statistical tests, one possible explanation could be that the fat distribution induced by phthalate exposure and subsequent insulin resistance may vary by gender [40]. Indeed, visceral versus subcutaneous fat mass is known to differ between men and women [41]. Another possibility lies in potential interactions of anti-androgenic effect by phthalates with modulation of insulin resistance [42] or glucose uptake [43]. Further investigation is needed to better understand these gender differences in phthalate-markers of diabetes risk associations.

Our analysis suggests racial/ethnic differences in markers of diabetes risk according to exposure levels of specific phthalates. Such racial/ethnic differences are unlikely to be explained solely by the higher phthalate levels in minorities, as the use of racial/ethnic-specific quartiles yielded similar associations to the use of population-specific quartiles. Also, the observed differences cannot be simply explained by the fat distribution hypothesis as postulated for gender differences, because a number of studies suggest non-Hispanic whites have higher visceral fat and liver fat than blacks and Mexican-Americans [41,44,45]. It is possible that the prevalence of certain susceptibility gene variants, such as Pro12Ala in PPAR-gamma gene that has been associated with type 2 diabetes risk and its intermediate traits [46,47], may vary by race/ethnicity [48]. However, these racial/ethnic differences need to be further explored.

The present exploratory study has several limitations. First, given the cross-sectional design of the study, we cannot make any causal interpretations for the observed associations. Second, the quickly metabolizing and excreting nature of phthalates [4,9], as well as the one-time collection of urine samples, does not provide reliable inference for the long-term exposure to phthalates [49]. Also, difference in urinary levels of phthalates metabolites may reflect differences in phthalate metabolism and excretion in addition to differences in phthalate exposure. Third, the existence of undiagnosed cases of diabetes [50] in our presumably metabolically normal participants might result in misclassification and overestimate the true associations, given that people with undiagnosed diabetes may have increased phthalate exposures through the use of medical devices and medications for co-morbid conditions [51], as well as greatly elevated levels of markers of diabetes risk. However, sensitivity analyses excluding participants with FBG > 126 mg/dL or hemoglobin A1c > 6.5% show negligible changes in the results. Phthalate exposures through medical devices and medications for other chronic diseases may also confound the association. Finally, we must interpret the results with caution, because statistically significant interactions were not noted despite consistent findings for racial/ethnic differences, likely due to increased degrees of freedom when phthalate exposures were assessed in quartiles and limited sample size in minority groups. Also, the possibility of chance findings cannot be ruled out as we did not adjust for multiple comparisons in the analysis.

The strength of this study includes an analysis of a large, representative U.S. population, in which we evaluated the association between 8 phthalate metabolites and 3 markers of diabetes risk. The pooling of 8-years of NHANES data provides better power for more elaborate assessment within gender and racial/ethnic strata. To our knowledge, this is one of the first studies to evaluate the association between phthalate metabolites and markers of diabetes risk evaluating effect modification by gender and race/ethnicity. Other strengths include our ability to account for a wide range of related covariates, including important risk factors and potential confounders available in NHANES 2001–2008. Furthermore, the median regression model, combined with exposure assessment in categories, was utilized to obtain easy-to-interpret and robust estimates that were less sensitive to potential outliers due to undiagnosed diabetes.

## Conclusions

In conclusion, the present exploratory study provides evidence for the potential impact of phthalates on glucose homeostasis and insulin resistance. The metabolic responses to this hypothesized phthalate effect, measured by FBG, fasting insulin and HOMA-IR, may vary by race/ethnicity and in some instances by gender. Future investigation should confirm our findings and look into the underlying mechanisms in both experimental and observational studies. If replicated in prospective studies and supported by mechanistic research, our results may suggest that reductions in concentrations of certain phthalate metabolites could improve glucose homeostasis and reduce type 2 diabetes risk, with a potentially stronger impact on more vulnerable subgroups of the population.

## Abbreviations

CDC: Centers for disease control and prevention; CI: Confidence interval; DEHP: Di-2-ethylhexyl phthalate; FBG: Fasting blood glucose; HOMA-IR: Homeostasis model assessment-estimated insulin resistance; LOD: Limit of detection; MBzP: Mono-benzyl phthalate; MCPP: Mono-(3-carboxypropyl) phthalate; MEHHP: Mono-(2-ethyl-5-hydroxyhexyl) phthalate; MEHP: Mono-(2-ethyl-hexyl) phthalate; MEOHP: Mono-(2-ethyl-5-oxohexyl) phthalate; MEP: Mono-ethyl phthalate; MiBP: Mono-isobutyl phthalate; MnBP: Mono-n-butyl phthalate; NHANES: National health and nutrition examination survey.

## Competing interests

The authors declare no competing interests.

## Authors’ contributions

TH participated in study concept and design, performed statistical analyses, interpreted the results and drafted the manuscript. ARS interpreted the results and critically revised the manuscript; EI interpreted the results and critically revised the manuscript. TJ conceived of the study, helped to draft the manuscript, interpreted the results and critically revised the manuscript. All authors read and approved the final manuscript.

## Supplementary Material

Additional file 1 Association of urinary phthalate metabolites with markers of diabetes risk by gender, NHANES 2001–2008. Click here for file

Additional file 2 Association of urinary phthalate metabolites with markers of diabetes risk by race/ethnicity, NHANES 2001–2008. Click here for file
